# Toward an Abolitionist Epidemiology of Displacement: Lessons From the United States on Border Detention of Migrants

**DOI:** 10.3389/ijph.2025.1608791

**Published:** 2025-07-15

**Authors:** Roberto Daniel Sirvent, Bilal Irfan

**Affiliations:** ^1^ Department of Global Health and Social Medicine, Harvard Medical School, Boston, MA, United States; ^2^ Center for Bioethics, Harvard Medical School, Boston, MA, United States; ^3^ Center for Surgery and Public Health, Brigham and Women’s Hospital, Boston, MA, United States; ^4^ Department of Neurology, University of Michigan Medical School, Ann Arbor, MI, United States; ^5^ Department of Epidemiology, University of Michigan School of Public Health, Ann Arbor, MI, United States

**Keywords:** migration and health, refugee health, detention, migrant, undocumented migrants

Rodolfo Saracci reminds us that the cascade of “exodus hazards” shadows displaced persons long before and long after a border is crossed, demanding public-health inquiry that is sensitive to the political economies that engineer flight and impede care [1]. Yet the North American literature has largely cordoned the health of forcibly displaced people into clinical inventories of trauma, infection, and “acculturation stress,” treating the border as an epidemiological baseline rather than an active site of exposure. We argue that detention of migrants itself, when understood as sitting at the lethal intersection of racism, carcerality, and colonial enclosure, must be repositioned from the backdrop to a central, quantifiable determinant of migrant morbidity and mortality. An abolitionist epidemiology that is grounded in anti-colonial traditions offers the conceptual and methodological tools required for that task.

The empirical record already indicts the U.S. detention archipelago. Drawing on death investigations released by U.S. Immigration and Customs Enforcement (ICE), Human Rights Watch and independent correctional-health experts found that in eight of fifteen detainee deaths reviewed (2015–2017), including a heart-attack patient denied urgent evaluation, a man whose congestive heart failure was repeatedly mismanaged, a pneumonia patient left 3 days with critically low oxygen, and a suicide that followed isolation despite known psychosis, substandard or delayed medical care directly contributed to the fatalities [2]. A recent qualitative study based on semi-structured phone interviews with sixteen immigrants who had spent a median of 11 months in ICE custody described how poor food, overcrowding, and prolonged confinement contributed to new or worsening cardiometabolic problems, and to pervasive symptoms of anxiety, insomnia, and suicidality; participants consistently perceived that the longer they were held, the more their physical and mental health deteriorated [3]. These individual-level findings echo facility-level audits exposing antibiotic shortages, absence of dialysis, and routine use of solitary confinement as a behavioral “treatment.” When epidemiologists abstract such data from the political conditions that reproduce them, they risk pathologizing migrants while naturalizing the carceral setting that produces illness.

That setting was never neutral. A brief history of Haitian refugee exclusion shows how the modern detention regime crystallized in the 1980s as a structurally anti-Black apparatus designed first to repel, then to immobilize and discredit, Caribbean-origin asylum seekers [4].

The Movement for Black Lives notes that Black migrants make up only about 7 percent of all non-citizens but roughly one in five people ordered deported on “criminal” grounds [5]. Its policy paper casts these numbers as evidence of a broader “war on Black migrants,” foregrounding the entwined technologies of policing, incarceration, and deportation that converge to produce disproportionate harm, including medical neglect and premature death, in Black migrant communities. Treating detention simply as an unfortunate administrative bottleneck rather than as an organized vector of racial violence underestimates its epidemiological force.

Citizenship is often prescribed as remedy, yet the promise of incorporation has never conferred immunity from structural neglect. Indigenous scholars of the Red Nation note that dispossession and border militarization co-evolved, and their “Red Deal” reframes health justice as land back, demilitarization, and communal stewardship [6]. Within that frame, disease clusters in detention are not system failures but predictable sequelae of a political economy that polices mobility while commodifying migrant labor. Public health metrics that valorize “pathways to citizenship” without interrogating the racial-capitalist soil into which that status is planted may inadvertently reproduce harm.

Externalized borders amplify those harms overseas. The Black Alliance for Peace documents how the United States Africa Command (AFRICOM) has expanded to fifty-three African states, coupling counter-migration surveillance with live-fire exercises that contaminate water tables and displace pastoralist communities [7]. Environmental epidemiology must therefore link transnational military emissions to cardiopulmonary disease patterns in host populations and to the forced migration they catalyze. Any serious account of migrant health that ends at the boundary fence undercounts the upstream toxicants of colonialism and imperialism.

Beyond detention, migrant health is shaped by the exploitative labor arrangements awaiting those who survive the border. Immigrant workers disproportionately occupy precarious jobs marked by low wages, hazardous exposures, and minimal protections, realities that can sometimes treat as background variables rather than central determinants of health. Housekeepers, construction laborers, and care workers, many of whom are Black, Brown, undocumented, or women, experience high rates of musculoskeletal injury, chemical exposure, and psychological distress [8]. In one Florida-based survey, nearly 60% of immigrant hotel housekeepers reported moderate to severe back and neck pain, sprains, and burns, with many suffering fingerprint loss from prolonged chemical contact despite glove use [8]. These injuries were strongly associated with poor-quality equipment and excessive workloads, yet the institutional indifference to these conditions reflects broader patterns of racial capitalism [8]. Similarly, the COVID-19 pandemic illuminated how low-wage immigrant workers, meatpackers, agricultural laborers, and janitorial staff bore disproportionate infection risk not due to biological vulnerability but because structural arrangements concentrated hazard in racialized, underprotected workplaces [9]. Epidemiologic studies show how race-based adjustments in occupational health metrics, such as pulmonary function thresholds, serve to systematically understate workplace harms and reduce industry accountability [9].

What, then, does an abolitionist epidemiology require? First, the method. Surveillance systems must disaggregate detention-related diagnoses, time-to-care intervals, and mortality by race, sex, and legal classification, moving beyond the generic “non-citizen” denominator. Second, praxis. Clinicians stationed at border encampments have begun to translate solidarity into refusal, for instance, by withholding medical clearance for deportations deemed clinically unsafe and by publicly documenting abuses in peer-reviewed forums [10]. Such acts illuminate the professional duty to disrupt structures that manufacture disease rather than merely palliate symptoms. Third, policy. Epidemiologists should lend quantitative backing to abolitionist campaigns that call for the closure, not reform, of detention facilities engaged in human rights violations, and the redirection of federal carceral budgets toward community-governed housing, food, and primary care infrastructures proven to shorten hospital stays and avert overdose deaths.

Public health as a discipline is challenging its scholars and contributors to grapple with displacement as both health exposure and analytic blind spot. Meeting that challenge demands a paradigm that refuses to exceptionalize the border while normalizing the violence behind it. Centering those most criminalized clarifies how abolition is not a metaphor but a measurable health intervention. In displacing detention from the clinical periphery to the epidemiological core, we may finally align public health with the emancipatory ambitions of those it purports to serve.
